# Podocyte-Specific Deletion of STAT3 in Krüppel-Like Factor 4–Related Experimental Podocytopathy

**DOI:** 10.1681/ASN.0000000841

**Published:** 2025-09-02

**Authors:** Yogesh Gowthaman, Chelsea C. Estrada, Joseph Kim, Yiqing Guo, Robert Bronstein, David J. Salant, John C. He, Vivette D. D’Agati, Sandeep K. Mallipattu

**Affiliations:** 1 Division of Nephrology, Department of Medicine, Stony Brook University, Stony Brook, New York; 2 Division of Nephrology, Department of Medicine, Northport Veterans Affairs Medical Center, Northport, New York; 3 Division of Nephrology, Department of Medicine, Boston University School of Medicine, Boston, Massachusetts; 4 Division of Nephrology, Department of Medicine, Icahn School of Medicine at Mount Sinai, New York, New York; 5 Department of Pathology and Cell Biology, Columbia University, New York, New York

**Keywords:** glomerular disease, glomerulopathy, podocyte

## Abstract

**Key Points:**

- Detrimental effects of Krüppel-like factor 4 knockdown in podocytes were eliminated with the inhibition of signal transducer and activator of transcription 3 (STAT3) signaling specifically in podocytes.
- Human kidney biopsies with renal vasculitis demonstrated a glomerular enrichment of STAT3 downstream genes, which negatively correlated with eGFR.
- Deconvolution of the bulk RNA-seq from Nephrotic Syndrome Study Network showed an enrichment of STAT3 downstream genes in podocytes as compared with other cell clusters.

**Background:**

Podocyte loss and parietal epithelial cell activation are features of subtypes of glomerulonephritis and FSGS. We recently reported that the podocyte-specific loss of *Krüppel-like factor 4* (*Klf4*^*ΔPod*^) triggers dysregulated glomerular signal transducer and activator of transcription 3 (STAT3) activation, podocyte loss with parietal epithelial cell activation and proliferation, leading to FSGS. Although pharmacologic systemic STAT3 inhibition attenuated this phenotype, it remains unclear whether the detrimental effects of *Klf4* loss are primarily a result of dysregulated STAT3 activation intrinsically in podocytes.

**Methods:**

Mice with the concurrent and conditional knockdown of *Stat3* and *Klf4* (*Klf4*^*ΔPod*^*Stat3*^*ΔPod*^) were generated and characterized. Expression arrays from kidney biopsies with various types of glomerular diseases, deposited in Nephroseq, were interrogated for glomerular expression of genes downstream of STAT3 signaling. Cell-specific modulation of STAT3 genes was determined using single-cell RNA sequencing–based proportional cell type deconvolution of bulk RNA-seq obtained from the Nephrotic Syndrome Study Network (NEPTUNE) FSGS and healthy controls.

**Results:**

*Klf4*^*ΔPod*^*Stat3*^*ΔPod*^ mice demonstrated no significant podocyte loss, parietal epithelial cell activation and proliferation, FSGS lesions, albuminuria, kidney dysfunction, and tubulointerstitial fibrosis and inflammation compared with the *Klf4*^*ΔPod*^ mice. *Klf4*^*ΔPod*^*Stat3*^*ΔPod*^ mice also exhibited less glomerular myofibroblasts (+*α*-smooth muscle actin) as compared with *Klf4*^*ΔPod*^ mice. Overall survival was restored in *Klf4*^*ΔPod*^*Stat3*^*ΔPod*^ mice as compared with *Klf4*^*ΔPod*^ mice. Interrogation of expression arrays from human kidney biopsies with renal vasculitis demonstrated a glomerular enrichment of genes involved in canonical STAT3 signaling as compared with healthy controls, which negatively correlated with eGFR. Deconvolution of the bulk RNA-seq data from NEPTUNE showed an enrichment of these STAT3 genes in podocytes as compared with other glomerular cell clusters.

**Conclusions:**

Collectively, these data demonstrate that inhibiting podocyte-specific STAT3 signaling was sufficient to counter the detrimental effects of *Klf4* loss in podocytes and prevented albuminuria, accelerated podocyte loss, activation and proliferation of parietal epithelial cells, FSGS lesions, and kidney failure.

## Visual Abstract

**Figure:**
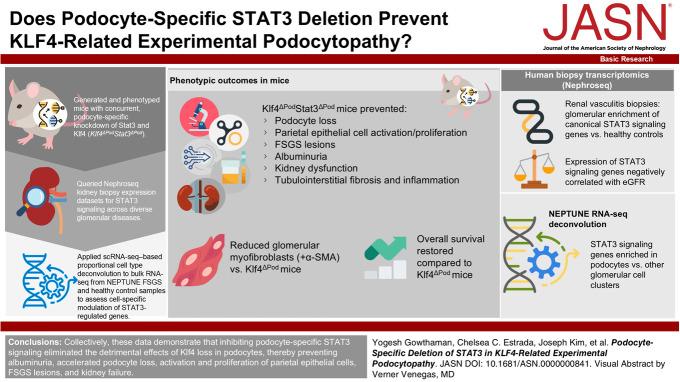

## Introduction

Podocytes are terminally differentiated, specialized visceral epithelial cells, which are integral components of the glomerular filtration barrier. The foot processes of adjacent podocytes interdigitate to form filtration slits bridged by slit diaphragm. Perturbations to this architecture can result in impaired filtration ability and leakage of proteins into the urine. Subtypes of acute GN and FSGS are characterized by initial podocyte loss leading to aberrant parietal epithelial cell activation and proliferation.^1,2^ These activated parietal epithelial cells contribute to the formation of pathologic crescents and pseudocrescents in subtypes of GN and FSGS, respectively, which ultimately contribute to glomerulosclerosis.^1–4^ The extent of crescentic lesions correlates with worse outcomes, as most patients with more than 50% crescentic glomeruli progress to kidney failure.^5^ Given the substantial morbidity and mortality associated with these diseases, a better understanding of the mechanisms involved in podocyte and parietal epithelial cells pathobiology is critical.

The Janus Kinase Signal Transducer and Activator of Transcription pathway is activated in a wide range of kidney diseases^6^ and has been demonstrated to be pathogenic in the development and progression of diseases characterized by podocyte injury.^7–9^ The podocyte-specific deletion of *Stat3* attenuated proteinuria and crescent formation after nephrotoxic serum (NTS) treatment, a transient murine model of GN with parietal epithelial cell activation.^7^ Furthermore, both global and podocyte-specific knockdown of *Stat3* attenuated glomerular injury in HIV-1 transgenic mice.^8,9^

Krüppel-like factors are a group of zinc-finger transcription factors, which are involved in diverse biologic processes, including differentiation, proliferation, pluripotency, and apoptosis.^10^ Krüppel-like factor 4 is a prodifferentiation transcription factor that functions as a negative regulator of cell proliferation.^11^ We recently reported the critical role of Krüppel-like factor 4 in maintaining podocyte homeostasis by inhibiting the activation of glomerular signal transducer and activator of transcription 3 (STAT3) signaling through direct protein–protein interaction.^12^ Specifically, mice with podocyte-specific knockdown of *Klf4* (*Klf4*^∆Pod^) exhibited rapid podocyte loss, parietal epithelial cell activation and proliferation with eventual glomerulosclerosis and kidney dysfunction (elevation in serum urea nitrogen and creatinine). In addition, 50% of the *Klf4*^∆Pod^ mice died by approximately 12 weeks of age and exhibited an increase in phospho-STAT3 (pSTAT3) expression (*i.e*., STAT3 activation) in several cell types in the kidney, including podocytes, parietal epithelial cells, tubular cells, and inflammatory cells.^12^ We also reported that treatment with a small-molecule inhibitor of STAT3 (S3I-201) in these *Klf4*^*∆Pod*^ mice partially improved albuminuria, podocyte injury, and parietal epithelial cell activation.^13^ However, it remains unclear whether the renoprotective effects of pharmacologic inhibition of STAT3 signaling in *Klf4*^*∆Pod*^ mice were due to its indirect systemic effects in multiple cell types as compared with the direct salutary effects in podocytes. In this study, we propose to test the hypothesis that the detrimental effects of *Klf4* loss in podocytes are primarily mediated through podocyte-specific dysregulation of STAT3 signaling. We also propose to test the extent that glomerular STAT3 signaling correlates with the progression of human glomerular disease.

## Methods

### Generation of Transgenic Mice

*Klf4*^*fl/fl*^ (FVB/N) and *Stat3*^*fl/fl*^ (FVB/N) mice were generated, as previously described,^9,12^ and crossed, separately, with mice expressing *Cre recombinase* under the control of the *Nphs2* promoter (B6.Cg-Tg [NPHS2-cre]295Lbh/J; The Jackson Laboratory). The resulting F2 generation resulted in the creation of *Klf4*^∆*Pod*^ mice and *Stat3*^∆*Pod*^ mice, respectively. *Klf4*^∆*Pod*^ mice and *Stat3*^∆*Pod*^ mice were then bred with each other to generate *Klf4*^∆*Pod*^*Stat3*^∆*Pod*^ mice. Mice were genotyped using a gel-based PCR, as previously reported,^12^ or by Transnetyx, Inc.

### Albuminuria Measurement

Urine albumin was measured with ELISA using a kit obtained from Bethyl Laboratory Inc. In the same set of samples, urine creatinine levels were determined using the Creatinine Colorimetric Assay Kit (Cayman), following the manufacturer's guidelines. The urine albumin excretion was calculated as the ratio of urine albumin-creatinine ratio.

### Measurement of Serum Urea Nitrogen and Creatinine Levels

Serum urea nitrogen levels were assessed using a colorimetric detection method (Arbor Assay) as per the manufacturer's instructions. Serum creatinine levels were determined through isotope dilution liquid chromatography–tandem mass spectrometry at the University of Alabama at Birmingham's O'Brien Core Center.

### Light Microscopy and Histology Quantification

Following perfusion with PBS, the mouse kidneys were fixed overnight in 10% phosphate-buffered formalin and transferred into 70% ethanol until histologic processing. Stony Brook University histology core facility embedded the kidney tissue in paraffin and prepared 3-*μ*m thick sections stained with hematoxylin and eosin and periodic acid–Schiff (MilliporeSigma). Quantification of histologic parameters in Table 1 was performed in blinded manner by the kidney pathologist (V.D. D’Agati).

**Table 1:** Quantification of histopathology in *Klf4*^*fl/fl*^*Stat3*^*fl/fl*^, *Klf4*^*ΔPod*^, and *Klf4*^*ΔPod*^*Stat3*^*ΔPod*^ mice groups

Variable	% Extracapillary Proliferation	% FSGS Lesions	% Tubular Injury	% Interstitial Inflammation	% Interstitial Fibrosis
LM control	0.0±0	0.0±0	0.0±0	0.0±0	0.0±0
*Klf4* ^*ΔPod*^	50±11^a^	37±10^a^	24±6^a^	30±5^a^^,^^b^	10±1^a^
*Klf4* ^*ΔPod*^ *Stat3* ^*ΔPod*^	0.0±0	0.0±0	0.0±0	0±0	0.0±0

Data are mean±SEM. *N*=5–6 mice/group. Kruskal–Wallis test. LM, littermate control.

a *P* < 0.01 (as compared with LM control and *Klf4*^*ΔPod*^*Stat3*^*ΔPod*^).

b *P* < 0.05 (as compared with *Klf4*^*ΔPod*^).

### Immunofluorescence and Quantification

The specimens underwent an initial baking phase of 60 minutes in an oven set at a temperature range of 55°C–60°C, followed by processing in accordance with established methods. The process involved deparaffinizing 3-*μ*m thick formalin-fixed and paraffin-embedded sections, with subsequent inactivation of endogenous peroxidase using H_2_O_2_. All antibodies and dilutions used are presented in Supplemental Table 1. Photographs of the slides were captured using a digital camera paired with a Nikon Eclipse i90 microscope. To assess the percentage of stained area, we used ImageJ 1.26 t software (National Institutes of Health, rsb.info.nih.gov/ij) and analyzed a minimum of 30 glomeruli from each sample.

### Isolation of Glomeruli from Mice for RNA Extraction

Mouse glomeruli were isolated using a previously described protocol.^12^ In brief, mice were perfused with PBS containing 2.5 mg/ml iron oxide and 0.1% BSA. The kidneys were subsequently removed, decapsulated, cut into 1-mm fragments, and digested in PBS supplemented with 1 mg/ml collagenase A and 100 U/ml deoxyribonuclease I. The digested tissue was filtered through a 100-μm cell strainer and centrifuged to collect the pellet. This pellet was resuspended in 1 ml of PBS, and glomeruli were separated using a magnet, and purity of the isolated glomeruli was verified under a microscope. Total RNA was extracted from the glomeruli using the RNAeasy kit (Qiagen).

### Real-Time PCR

Total RNA was initially extracted from isolated glomeruli or kidney cortex samples using the RNeasy kit (Qiagen). Subsequently, cDNA was prepared using the SuperScript IV VILO Master Mix (Life Technologies). The resulting diluted cDNA was then amplified in triplicate using PowerUp SYBR quantitative PCR Master Mix on an ABI QuantStudio 3 instrument (Applied Biosystems). Primer sequences were designed using National Center for Biotechnology Information/Primer-BLAST and presented in Supplemental Table 2. Subsequently, the data were normalized to housekeeping genes (*Beta Actin* [*Actb*]) and presented as a fold change increase relative to the mean normalized mRNA expression from the control group, which was calculated using the 2^∆∆CT^ method.

### Analysis of Human Biopsy Sequencing Data

R package pheatmap was used to generate the hierarchically clustered heatmap of the NephroSeq dataset^14^ stemming from a STAT3 gene target list. A matrix of log_2_ fold change values was used as the input for the analysis with the following counts of samples included for control and each disease state: control (21), diabetic kidney disease (DKD; 12), FSGS (25), hypertension (15), IgA nephropathy (27), lupus nephritis (32), minimal change disease (14), and renal vasculitis (23) (Supplemental Table 3). The individual samples for each disease state were collapsed into single heatmap column blocks. The left-sided dendrogram is also included to display lineage relationships between the different genes across all control and disease samples.

Bulk RNA-seq cell type deconvolution analysis was performed with Multi-subject Single Cell deconvolution (MuSiC2)^15^ using kidney single-cell RNA sequencing (scRNA-seq) datasets as a reference. In preparation for MuSiC2 deconvolution GSE254957,^16^ bulk RNA-seq dataset gene expression matrices corresponding to eight living donor controls and 25 FSGS patients were retrieved from the Gene Expression Omnibus and converted into two merged bulk control and case matrices. We used a merged scRNA-seq reference dataset originating from the Gene Expression Omnibus repository GSE185948,^17^ representing five scRNA-seq datasets from healthy kidney cortex samples. Preprocessing of the GSE185948 data included conversion of ENSEMBL IDs to common gene symbols, matching those found in the bulk data matrices. To reduce overlapping gene signatures in highly related clusters, which would obscure accurate cell type proportion calls during deconvolution, we merged the following clusters: PT1 and PT2 (proximal tubule), TAL1 and TAL2 (thick ascending limb), CNT_PC and distal convoluted tubule (connecting tubule/distal convoluted tubule), and intercalated cell Type A and intercalated cell Type B (intercalated cell). In addition, in the bulk/control matrices, we removed any 0, not applicable, or negative values. For deconvolution, we used the MuSiC2 T-statistics approach to detect cell type–specific differentially expressed genes, with default settings apart from a STAT3 downstream target input gene list. We proceeded to plot the resultant proportions with a ggplot2 jitter plot (geom_jitter).

### Statistical Analysis

We used a range of statistical techniques to analyze our data based on number of groups and distribution. Sample sizes for mice studies were calculated based on estimated effect sizes from our previous studies of *Klf4*^∆*Pod*^ mice.^12^ All data were first assessed for normality, and then, parametric or nonparametric tests were used, as appropriate. For normally distributed data with two groups, we used a *t* test and in cases where comparisons involved more than two groups, we used a two-way ANOVA, followed by a Tukey post-test for further analysis. For non-normally distributed data, the Mann–Whitney *U* test was applied for comparing continuous data between two groups. We used a Pearson correlation test to analyze the relationship between two variables. The correlation coefficient, *r*, was computed and quantified the direction and magnitude of the correlation. Simple linear regression was used to identify and visualize linear relationships between two variables. Each experiment's statistical test is detailed in the figure legends, and the results were presented as the mean±SEM. All experiments were performed at least three times, and we have included representative results. Statistical significance was determined with a *P* value threshold of < 0.05. All statistical analyses were conducted using GraphPad Prism 10.0.

### Study Approval

All animal studies conducted were approved by the Stony Brook University Animal Institute Committee. National Institutes of Health Guide for the Care and Use of Laboratory Animals was followed strictly.

## Results

### Combined Loss of Podocyte-Specific *Klf4* and *Stat3* Prevents the Development of Glomerulosclerosis and Kidney Dysfunction in Podocyte-Specific *Klf4* Knockout Mice

**Figure 1:**
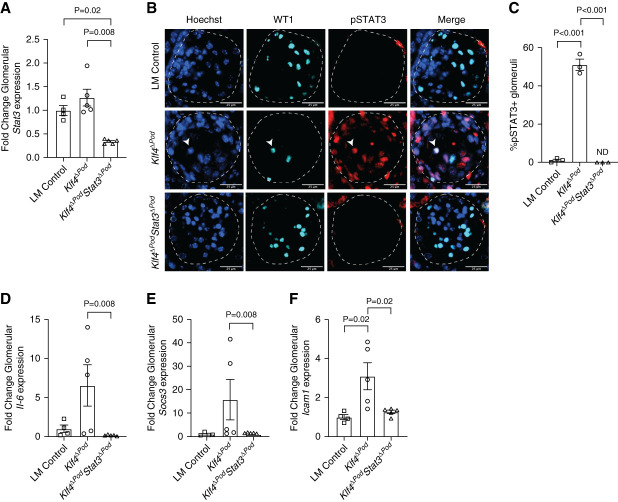
**Stat3 expression and activation is reduced in *Klf4***^***ΔPod***^***Stat3***^***ΔPod***^ **mice.** (A) Relative *Stat3* glomerular mRNA expression; *N*=4–5 mice/group; Mann–Whitney test. (B) Representative images of glomerular pSTAT3 and WT1 coimmunostaining; dashed white lines indicate glomeruli area; arrowhead indicates colocalization of WT1 and pSTAT3; scale bars=25 *µ*m. (C) Quantification of percentage of glomeruli with pSTAT3+ cells; *N*=3 mice/group and 20 glomeruli/mouse; Mann–Whitney *U* test. (D–F) Relative *Il-6*, *Socs3*, *Icam-1* glomerular mRNA expression; *N*=4–5 mice/group; Mann–Whitney *U* test. *Icam-1*, *intracellular adhesion molecule 1*; LM, littermate control; pSTAT3, phospho-STAT3; *Socs3*, *suppressor of cytokine signaling 3*; STAT3, signal transducer and activator of transcription 3; WT1, Wilms tumor 1.

We previously demonstrated that the podocyte-specific loss of *Klf4* (*Klf4*^*ΔPod*^) results in podocyte loss, parietal epithelial cell activation and proliferation, with FSGS lesions in mice.^12^ We also reported that this might be, in part, due to a loss of KLF4-mediated pSTAT3 inhibition, resulting in dysregulated STAT3 signaling.^12^ To test whether the detrimental effects in *Klf4*^*ΔPod*^ mice were due primarily to failed pSTAT3 inhibition, specifically in podocytes, *Stat3*^*fl/fl*^ and *Klf4*^*fl/fl*^ mice were bred with *Podocin-Cre* mice to generate mice with the combined podocyte-specific knockdown of *Stat3* and *Klf4* (*Klf4*^*ΔPod*^*Stat3*^*ΔPod*^) and were compared with the *Klf4*^*ΔPod*^ mice and *Klf4*^*fl/fl*^*Stat3*^*fl/fl*^ (littermate control) mice. We previously generated and reported podocyte-specific *Stat3* knockdown (*Stat3*^*ΔPod*^) mice using the same *Cre recombinase* under the similar *Nphs2* promoter.^9^ These *Stat3*^*ΔPod*^ mice were viable and fertile with no kidney abnormalities.^7,9^ Real-time PCR of isolated glomeruli confirmed the decrease in *Stat3* expression in *Klf4*^*ΔPod*^*Stat3*^*ΔPod*^ mice as compared with *Klf4*^*ΔPod*^ and littermate controls (Figure 1A). Coimmunostaining with Wilms tumor 1 (WT1) and pSTAT3 showed colocalization in *Klf4*^*ΔPod*^ mice as well as nonpodocyte expression for pSTAT3 (Figure 1B). We also observed that the increase in glomerular nuclear pSTAT3 expression (*i.e*., STAT3 activation) in *Klf4*^*ΔPod*^ mice was eliminated in the *Klf4*^*ΔPod*^*Stat3*^*ΔPod*^ mice (Figure 1C). In addition, the previously reported increase in expression of the downstream STAT3 signaling genes in isolated glomeruli from *Klf4*^*ΔPod*^ mice^12^ (*IL-6*, *suppressor of cytokine signaling 3*, and *intracellular adhesion molecule 1*) returned to baseline in *Klf4*^*ΔPod*^*Stat3*^*ΔPod*^ mice (Figure 1, D–F).

**Figure 2:**
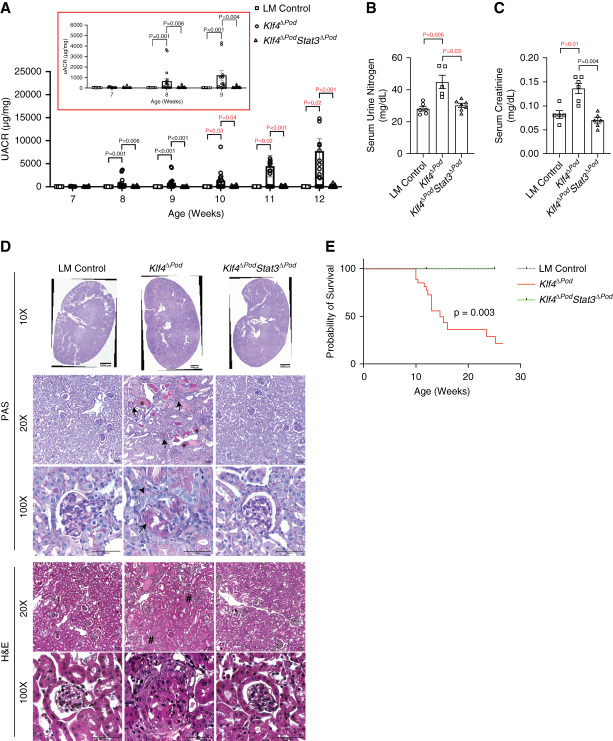
***Klf4***^***ΔPod***^***Stat3***^***ΔPod***^ **mice demonstrate less kidney injury and improved survival.** (A) UACR from 7 to 12 weeks; *N*=13–25 mice/group; two-way ANOVA (indicated with red text); Mann–Whitney *U* test (indicated with black text). Serum (B) urea nitrogen and (C) creatinine at 12 weeks; *N*=5–7 mice/group; Kruskal–Wallis test (indicated with red text); Mann–Whitney *U* test (indicated black text). (D) Representative whole kidney scans of PAS staining (×20, scale bars=1000 *µ*m) and higher-power PAS and H&E staining (×20 and ×100, scale bars=50 *µ*m). Representative images are shown to depict extracapillary proliferation (black and white arrowhead), glomerulosclerosis (arrow), protein casts (*), tubular dilation and interstitial inflammation (#). (E) Survival curves are shown for each group until age 27 weeks; *N*=19–26 mice/group; log-rank (Mantel–Cox test). H&E, hematoxylin and eosin; PAS, periodic acid–Schiff; UACR, urine albumin to creatinine ratio.

*Klf4*^*ΔPod*^ mice exhibited a significant increase in albuminuria starting at 8 weeks of age, which was not observed in either *Klf4*^*ΔPod*^*Stat3*^*ΔPod*^ mice or littermate controls (Figure 2A). Similarly, both serum urea nitrogen (Figure 2B) and creatinine (Figure 2C) were significantly increased in *Klf4*^*ΔPod*^ mice as compared with both *Klf4*^*ΔPod*^*Stat3*^*ΔPod*^ mice and littermate controls. Periodic acid–Schiff and hematoxylin and eosin revealed that *Klf4*^*ΔPod*^ mice developed FSGS lesions with pseudo-crescent formation and protein casts, which were absent in the *Klf4*^*ΔPod*^*Stat3*^*ΔPod*^ mice (Figure 2D and Table 1). In addition, *Klf4*^*ΔPod*^ exhibited an increase in both tubulointerstitial inflammation and fibrosis compared with littermate controls, which was markedly attenuated in *Klf4*^*ΔPod*^*Stat3*^*ΔPod*^ mice (Figure 2D and Table 1). Finally, <50% of the *Klf4*^*ΔPod*^ mice survived to 25 weeks of age, while all *Klf4*^*ΔPod*^*Stat3*^*ΔPod*^ mice survived through the entire experimental period (Figure 2E). In all, the conditional knockdown of *Stat3* in podocytes of *Klf4*^*ΔPod*^ mice prevented the development of kidney injury and preserved overall survival as compared with *Klf4*^*ΔPod*^ mice.

### *Klf4*^*ΔPod*^*Stat3*^*ΔPod*^ Preserved Podocyte Number and Parietal Epithelial Cell Quiescence in Mice

**Figure 3:**
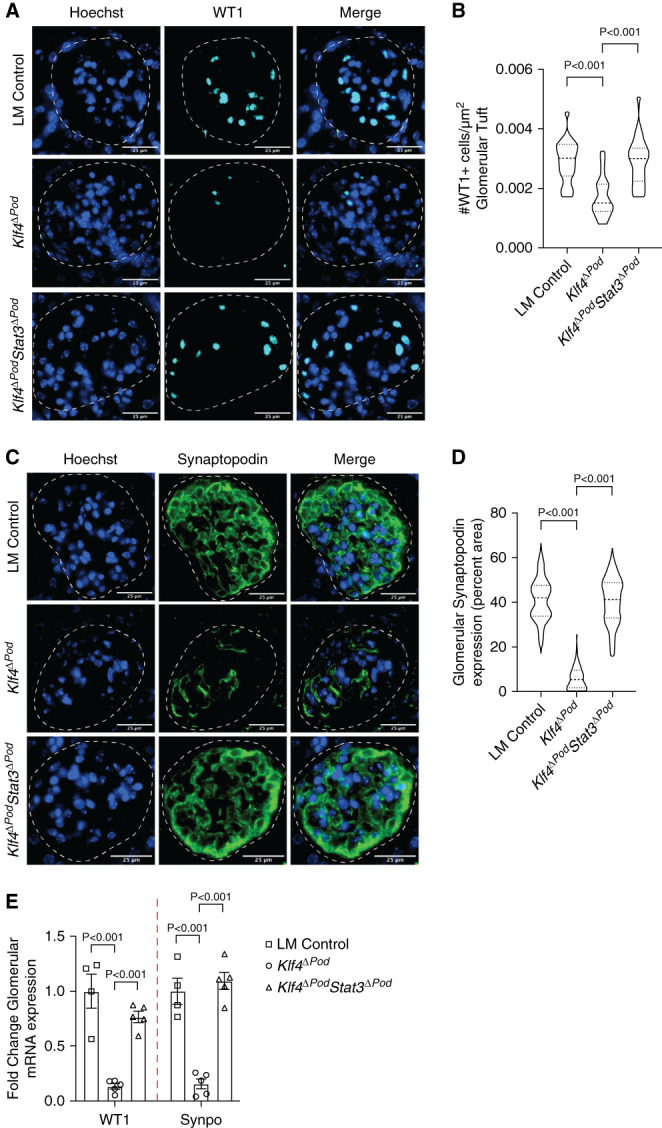
***Klf4***^***ΔPod***^***Stat3***^***ΔPod***^ **maintains podocyte number and actin cytoskeleton.** (A and B) Representative images of immunostaining for WT1 and quantification of the number of WT1+ cells per glomerular cross-sectional area; dashed white lines indicate glomeruli area, scale bars=50 *µ*m; *N*=3 mice/group and 20 glomeruli/mouse; Kruskal–Wallis test. (C and D) Representative images of immunostaining of Synaptopodin and quantification of percent area stained in glomeruli; dashed white lines indicate glomeruli area, scale bars=25 *µ*m; *N*=3 mice/group and 20 glomeruli/mouse; Kruskal–Wallis test. (E) Relative *Wt1* and *Synaptopodin* (*Synpo*) glomerular mRNA expression, *N*=4–5 mice/group, two-way ANOVA.

Since *Klf4*^*ΔPod*^*Stat3*^*ΔPod*^ mice prevented the development of significant FSGS lesions and albuminuria as compared with *Klf4*^*ΔPod*^ mice, we evaluated markers of podocyte health (podocyte number and actin cytoskeleton). Previously, we reported that the loss of *Klf4* in podocytes triggered cell cycle reentry, but resulted in mitotic catastrophe, and subsequent cell detachment and death.^12^ Immunofluorescence staining for WT1, a specific marker of differentiated podocytes, and quantification of # of WT1+ cells per glomeruli, showed that podocyte number was preserved in *Klf4*^*ΔPod*^*Stat3*^*ΔPod*^ as compared with *Klf4*^*ΔPod*^ mice (Figure 3, A and B). Similarly, immunofluorescence staining for Synaptopodin, a critical actin cytoskeleton protein in podocytes,^18^ and quantification of % glomerular Synaptopodin expression, confirmed that actin cytoskeleton was preserved in *Klf4*^*ΔPod*^*Stat3*^*ΔPod*^ as compared with *Klf4*^*ΔPod*^ mice (Figure 3, C and D). Real-time PCR for *Wt1* and *Synaptopodin* in isolated glomeruli from these mice also validated these findings (Figure 3E).

**Figure 4:**
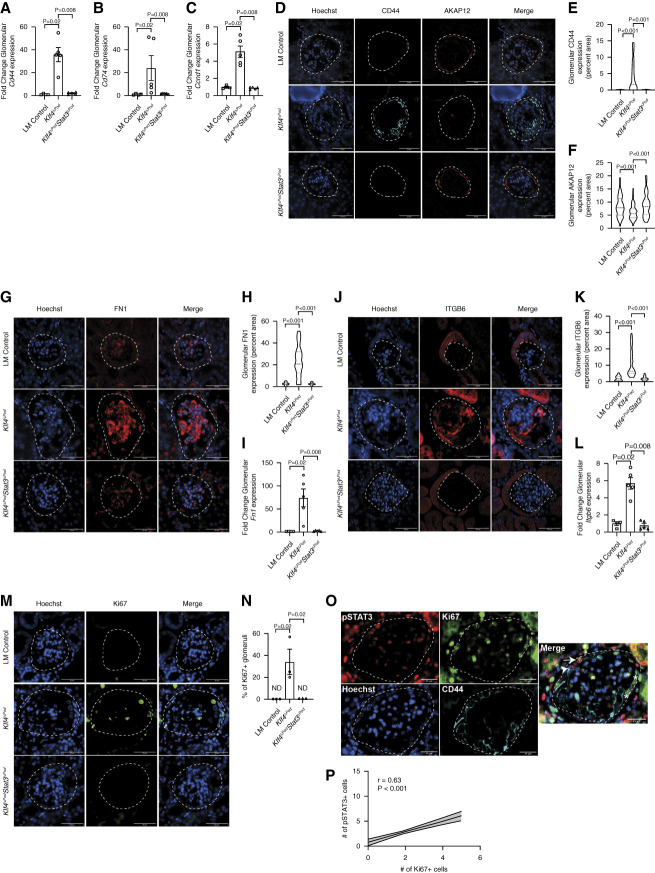
***Klf4***^***ΔPod***^***Stat3***^***ΔPod***^ **maintains parietal epithelial cell quiescence.** (A–C) Relative *Cd44*, *Cd74*, and *Ccnd1* glomerular mRNA expression; *N*=4–5 mice/group; Mann–Whitney *U* test. (D) Representative images of coimmunostaining for CD44 and AKAP12; dashed white lines indicate glomeruli area (scale bars=50 *µ*m) and (E and F) quantification of percent glomerular area stained; *N*=3 mice/group and 20 glomeruli/mouse; Kruskal–Wallis test. (G) Representative images of immunostaining for FN1; dashed white lines indicate glomeruli area (scale bars=50 *µ*m) and (H) quantification of percent glomerular area stained; *N*=3 mice/group and 20 glomeruli/mouse; Kruskal–Wallis Test. (I) Relative mRNA expression of glomerular *Fn1*; *N*=4–5 mice/group; Mann–Whitney *U* test. (J) Representative images of immunostaining for ITGB6; dashed white lines indicate glomeruli area (scale bars=50 *µ*m) and (K) quantification of percent glomerular area stained; *N*=3 mice/group and 20 glomeruli/mouse; Kruskal–Wallis test. (L) Relative mRNA expression of glomerular *Itgb6*; *N*=4–5 mice/group; Mann–Whitney *U* test. (M) Representative images of immunostaining for KI67; dashed white lines indicate glomeruli area (scale bars=50 *µ*m) and (N) quantification of percent of KI67+ glomeruli; *N*=3 mice/group and 20 glomeruli/mouse; Kruskal–Wallis test. (O) Coimmunostaining of pSTAT3, CD44, and KI67 in *Klf4*^*ΔPod*^ mice; dashed white lines indicate glomeruli area (scale bars=25 *µ*m). Representative images show coexpression of pSTAT3, KI67, and CD44 (arrow), coexpression of pSTAT3 and CD44 (arrowhead), and coexpression of CD44 and KI67 (*). (P) Quantification and correlation of the number of pSTAT3+ cells and KI67+ cells present in the same glomeruli in *Klf4*^*ΔPod*^ mice; *N*=3 mice/group and 20 glomeruli/mouse; plotted to visualize correlation and analyzed by a Pearson correlation test and simple linear regression; best-fit 95% confidence intervals are shown. AKAP12, A-kinase anchor protein 12; FN1, fibronectin 1.

To assess the extent of parietal epithelial cell activation, we initially measured the mRNA expression of *Cluster of Differentiation 44* (*Cd44*), *Cluster of Differentiation 74* (*Cd74*), and *Cyclin D1* (*Ccnd1*)^19,20^ in isolated glomeruli from all groups. *Cd44*, *Cd74,* and *Ccnd1* expression were all attenuated in *Klf4*^*ΔPod*^*Stat3*^*ΔPod*^ mice as compared with *Klf4*^*ΔPod*^ mice (Figure 4, A–C). In addition, coimmunofluorescence staining for CD44, a cell surface glycoprotein marker of activated parietal epithelial cells,^19^ and A-kinase anchor protein 12 src-suppressed C kinase substrate a multivalent scaffolding protein specific to quiescent parietal epithelial cells that sequesters Ccnd1 and prevents parietal epithelial cell proliferation,^20^ showed that glomerular CD44 expression was significantly decreased, and glomerular A-kinase anchor protein 12 expression was significantly increased, in *Klf4*^*ΔPod*^*Stat3*^*ΔPod*^ mice as compared with *Klf4*^*ΔPod*^ mice (Figure 4, D–F). We next evaluated the glomerular expression of the extracellular matrix protein, fibronectin 1 (FN1), as we previously demonstrated that *Fn1* was increased in isolated glomeruli from *Klf4*^*ΔPod*^ mice as well as in the podocyte secretome from *KLF4-shRNA* human podocytes.^13^ Glomerular FN1 expression was reduced in *Klf4*^*ΔPod*^*Stat3*^*ΔPod*^ mice as compared with *Klf4*^*ΔPod*^ mice by immunofluorescence staining and in real-time PCR of isolated glomeruli (Figure 4, G–I). We also previously demonstrated that *α*V*β*6 was upregulated in activated parietal epithelial cells, through the binding of FN1 with the R-G-D sequence.^13^ Immunofluorescence staining and real-time PCR for ITGB6 showed a reduction in glomerular expression in *Klf4*^*ΔPod*^*Stat3*^*ΔPod*^ mice as compared with *Klf4*^*ΔPod*^ mice (Figure 4, J–L). Finally, to evaluate for the extent of glomerular epithelial cells proliferation, we performed immunofluorescence staining for KI67 and demonstrated reduced cell cycle reentry (% of glomeruli with +KI67 cells) in *Klf4*^*ΔPod*^*Stat3*^*ΔPod*^ mice as compared with *Klf4*^*ΔPod*^ mice cells (Figure 4, M and N). Furthermore, coimmunofluorescence staining for pSTAT3, KI67, and CD44 to evaluate the relationship between STAT3 activation, cell cycle reentry, and parietal epithelial cell activation revealed that CD44 coexpressed with both KI67 and pSTAT3 independently, as well as scattered coexpression of all three proteins together in *Klf4*^*ΔPod*^ mice (Figure 4O). To further define the relationship between cell cycle reentry and STAT3 activation, we quantified individual glomeruli for number of KI67+ and pSTAT3+ cells and showed that the # of KI67+ cells per glomeruli correlated with the # of pSTAT3+ cells per glomeruli in *Klf4*^*ΔPod*^ mice (Figure 4P). These data suggest that the podocyte-specific knockdown of *Stat3* in *Klf4*^*ΔPod*^ mice prevented podocyte loss as well as STAT3-dependent parietal epithelial cell activation and cell cycle reentry in podocytes and neighboring parietal epithelial cells.

### *Klf4*^*ΔPod*^*Stat3*^*ΔPod*^ Mice Showed a Reduction in Tubulointerstitial Fibrosis and Inflammation and Accumulation of Glomerular Myofibroblasts in Mice

**Figure 5:**
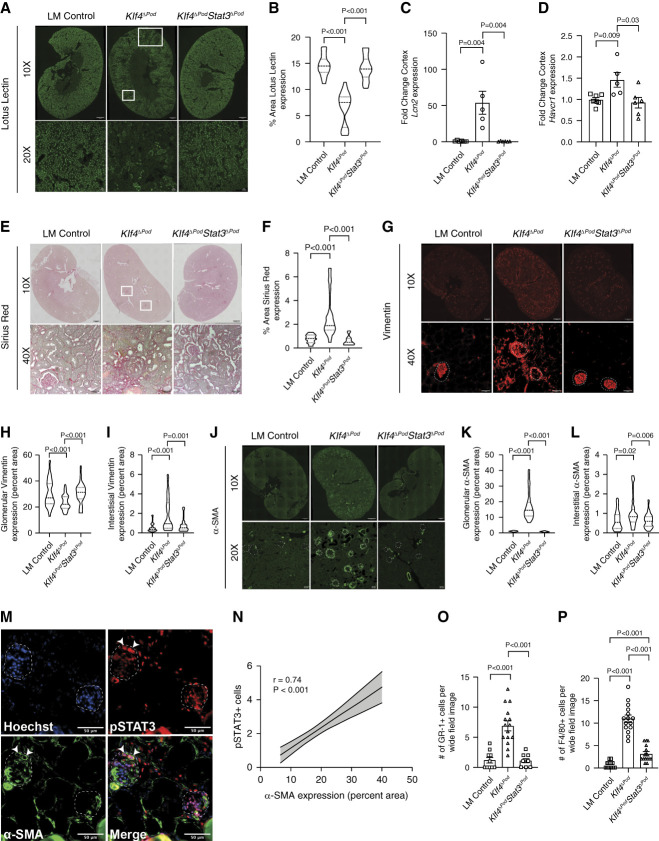
***Klf4***^***ΔPod***^***Stat3***^***ΔPod***^ **mice are protected against tubular injury and interstitial fibrosis and inflammation.** (A) Representative whole kidney scans (×10, scale bars=1000 *µ*m) and images (×20, scale bars=50 *µ*m) of LL (white box—examples of reduced LL expression) and (B) quantification of percentage of area stained; *N*=3 mice/group with 20 images per mouse; Kruskal–Wallis test. (C and D) Relative mRNA expression of *Lcn2* and *Havcr1* in kidney cortex; *N*=4–5 mice/group; Mann–Whitney *U* test. (E) Representative whole kidney scans (×10, scale bars=1000 *µ*m) and images (×40, scale bars=50 *µ*m) of Sirius Red (white box—examples of areas with reduced Sirius Red expression) and (F) quantification of percentage of area stained; *N*=3 mice/group with 20 images per mouse; Kruskal–Wallis test. (G) Representative whole kidney scans (×10, scale bars=1000 *µ*m) and images (×40, dashed white lines indicate glomeruli area, scale bars=50 *µ*m) of Vimentin with quantification of (H) percentage of glomerular area stained and (I) percentage of interstitial area stained (*N*=3 mice/group with 20 images per mouse); Kruskal–Wallis *U* test. (J) Representative whole kidney scans (×10, scale bars=1000 *µ*m) and images (×20, dashed white lines indicate glomeruli area, scale bars=50 *µ*m) of *α*-SMA and quantification of (K) percentage of glomerular area stained, and (L) percentage of interstitial area stained (*N*=3 mice/group with 20 glomeruli per mouse); Kruskal–Wallis test. (M) Coimmunostaining of pSTAT3 and *α*-SMA in *Klf4*^*ΔPod*^ mice; dashed white lines indicate glomeruli area, arrowhead shows coexpression of pSTAT3 and *α*-SMA. (N) Quantification and correlation of the number of pSTAT3+ cells and *α*-SMA cells present in the same glomeruli in *Klf4*^*ΔPod*^ mice; *N*=3 mice and 20 glomeruli/mouse plotted to visualize correlation and analyzed by a Pearson correlation test and simple linear regression; best-fit 95% confidence intervals are shown. (O) Quantification of number of GR-1+ and (P) F4/80+ cells per 20× wide field image, *N*=3 mice/group with five images/mouse; Kruskal–Wallis test. *α*-SMA, *α*-smooth muscle actin; GR-1, granulocyte receptor-1; LL, lotus lectin.

To test whether the salutary effects of concurrent *Stat3* knockdown in *Klf4*^*ΔPod*^ mice were restricted to the glomerulus, we initially conducted immunofluorescence staining for lotus lectin, marker of intact proximal tubule brush border.^21^ The expression and apical distribution of lotus lectin staining was restored in *Klf4*^*ΔPod*^*Stat3*^*ΔPod*^ mice as compared with *Klf4*^*ΔPod*^ mice (Figure 5, A and B). In addition, *Lcn2* (*Lipocalin-2* [encodes for neutrophil gelatinase–associated lipocalin] and *Havcr1* [*Hepatitis A virus cellular receptor 1* encodes for kidney injury molecule-1]) mRNA expression were also reduced in the *Klf4*^*ΔPod*^*Stat3*^*ΔPod*^ mice as compared with *Klf4*^*ΔPod*^ mice (Figure 5, C and D). *Klf4*^*ΔPod*^ mice showed an increase in % of interstitial Picrosirius (Sirius) Red, a marker of collagen 1 and 3,^22^ which was reduced in *Klf4*^*ΔPod*^*Stat3*^*ΔPod*^ mice (Figure 5, E and F). Semiquantitative scoring by the kidney pathologist in a blinded fashion also showed an improvement in the extent of tubular injury and interstitial fibrosis in *Klf4*^*ΔPod*^*Stat3*^*ΔPod*^ mice as compared with *Klf4*^*ΔPod*^ mice (Table 1). In the glomerular compartment, Vimentin serves as an intermediate filament protein in podocyte major foot processes.^23^ Immunofluorescence staining for Vimentin demonstrated a reduction in glomerular expression in *Klf4*^*ΔPod*^ mice as compared with *Klf4*^*ΔPod*^*Stat3*^*ΔPod*^ mice (Figure 5, G and H), a pattern previously reported in kidney biopsies from patients with FSGS.^24^ Conversely, Vimentin is typically not expressed in the interstitial compartment, and an increase in interstitial expression has been associated with increased fibrosis. *Klf4*^*ΔPod*^ mice exhibited higher expression of interstitial Vimentin, which was restored in *Klf4*^*ΔPod*^*Stat3*^*ΔPod*^ mice (Figure 5, G and I). Furthermore, immunofluorescence staining for *α*-smooth muscle actin (*α*-SMA), a marker of myofibroblasts and previously been reported to correlate with glomerulosclerosis and severity of tubulointerstitial fibrosis,^25^ demonstrated an increase in interstitial and glomerular myofibroblasts in *Klf4*^*ΔPod*^ mice, which was attenuated in *Klf4*^*ΔPod*^*Stat3*^*ΔPod*^ mice (Figure 5, J–L).

To evaluate the relationship between glomerular *α*-SMA and STAT3 activation, we performed coimmunofluorescence staining for *α*-SMA and pSTAT3 and observed some coexpression within the glomerulus in *Klf4*^*ΔPod*^ mice (Figure 5M). Quantification demonstrated that the number of pSTAT3+ cells per glomerulus directly correlated with % glomerular *α*-SMA staining per glomerulus (Figure 5N).

Finally, semiquantitative scoring by the kidney pathologist also showed a significant increase in interstitial inflammation in *Klf4*^*ΔPod*^ mice, which was attenuated in *Klf4*^*ΔPod*^*Stat3*^*ΔPod*^ mice (Table 1). Immunofluorescence staining for granulocyte receptor-1, marker of leukocytes,^26^ and F4/80, marker of macrophages,^27^ also showed a reduction in # of Gr1+ leukocytes and F4/80^+^ macrophages per high power field in *Klf4*^*ΔPod*^*Stat3*^*ΔPod*^ mice as compared with *Klf4*^*ΔPod*^ mice (Figure 5, O and P). These data suggest that the podocyte-specific knockdown of *Stat3* in *Klf4*^*ΔPod*^ mice attenuated the accumulation of glomerular myofibroblasts as well as the development of tubular injury and interstitial fibrosis and inflammation.

### Glomerular Enrichment of STAT3 Target Genes Negatively Correlated with eGFR in Human Kidney Biopsies

**Figure 6:**
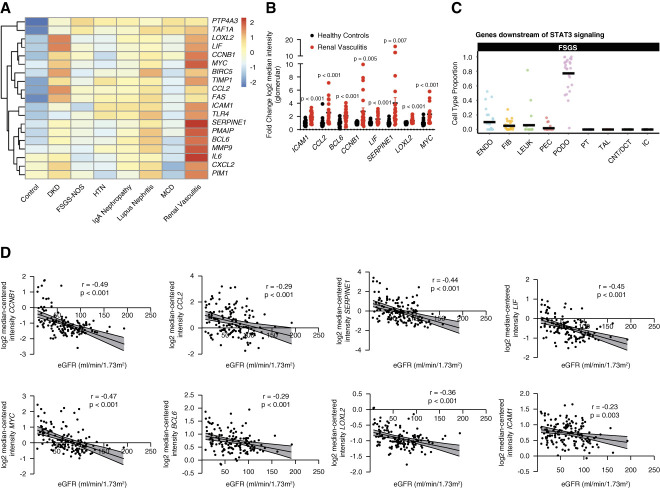
**Downstream STAT3 target gene expression in human glomerular diseases.** (A) Heatmap and hierarchical clustering of previously reported downstream STAT3 target genes from microdissected glomeruli from human kidney biopsies across various glomerular diseases in *Nephroseq*.^14^ (B) Glomerular expression of STAT3 target genes (*ICAM1*, *CCL2*, *BCL6*, *CCNB1*, *LIF*, *SERPINE1*, *LOXL2*, *MYC*) from kidney biopsies with renal vasculitis and healthy living donors; unpaired two-tailed *t* test). (C) Jitter plot of expression of STAT3 downstream target genes by cell type. (D) Glomerular expression of downstream STAT3 target genes in microdissected glomeruli from human kidney biopsies with renal vasculitis, FSGS-NOS, DKD, lupus nephritis, and IgA nephropathy plotted relative to eGFR. Analyzed by a Pearson correlation test and simple linear regression; best-fit 95% confidence intervals are shown. CNT/DCT, connecting tubule/distal convoluted tubule; DKD, diabetic kidney disease; Endo, endothelial; Fib, fibroblast; FSGS-NOS, focal segmental glomerulosclerosis- not otherwise specified; IC, intercalated cell; Leuk, leukocyte; PEC, parietal epithelial cell; Podo, podocyte; PT, proximal tubule; TAL, thick ascending limb.

To further evaluate the role of STAT3 signaling in human glomerular diseases, we interrogated previously reported arrays of microdissected glomeruli from kidney biopsies with a range of glomerular diseases in a *Nephroseq* database^14^ (Supplemental Table 3). Using hierarchical clustering, we initially observed an enrichment of several key downstream STAT3 targets in the glomeruli from kidney biopsies with renal vasculitis and to a lesser extent DKD and lupus nephritis as compared with other glomerular diseases (Figure 6A). We further examined the expression of the highest expressed STAT3 target genes specifically in renal vasculitis and found them to be significantly increased as compared with healthy controls (Figure 6B). To assess the degree of glomerular cell specificity in this STAT3 activation in glomerular disease (Figure 6A), we used single-cell RNA-seq–based proportional cell type deconvolution of bulk RNA-seq data obtained from the Nephrotic Syndrome Study Network (NEPTUNE) of 25 bulk RNA-seq datasets from biopsies with FSGS and eight healthy controls. The overall deconvolution demonstrated an enrichment of STAT3 signaling cascades in human podocytes as compared with other glomerular cells (Figure 6C). Finally, the glomerular expression of these key STAT3 target genes (Figure 6B) negatively correlated with eGFR (Figure 6D), suggesting the potential detrimental effect of activation of glomerular STAT3 signaling to kidney function. In all, enrichment of STAT3 downstream signaling was observed in the glomeruli and, specifically podocytes, in human glomerular disease and negatively correlated with eGFR.

## Discussion

The activation of STAT3 signaling has been identified as pathogenic across glomerular diseases, including lupus nephritis,^28^ DKD,^29^ collapsing FSGS,^30^ renal vasculitis,^12^ and others, while its inhibition, either directly or indirectly, has been shown to be protective.^13,31^ Furthermore, the podocyte-specific deletion of *Stat3* attenuated glomerular injury in murine models of collapsing FSGS^9^ and crescentic GN.^7^ KLF4 is critical for podocyte differentiation, and loss of KLF4 results in dysregulated STAT3 signaling in podocytes, leading to cell cycle reentry, and subsequent mitotic catastrophe.^12^ We previously demonstrated that the pharmacologic inhibition of STAT3 signaling attenuated albuminuria and glomerular injury in *Klf4*^*ΔPod*^ mice.^12,13^ However, it was unclear whether this renoprotective effect of pharmacologic inhibition of STAT3 signaling in *Klf4*^*∆Pod*^ mice is due to its direct inhibition in podocytes versus its systemic inhibition in other cell types. Here, we closely define the detrimental role of podocyte-specific *Stat3* in *Klf4*^*ΔPod*^ mice by showing that the conditional knockdown of *Stat3* specifically in podocytes in *Klf4*^*ΔPod*^ mice is sufficient to mitigate the development of albuminuria, rapid podocyte loss, parietal epithelial cell activation and proliferation, FSGS lesions, and kidney failure. Furthermore, this sole knockdown of *Stat3* in podocytes restored overall survival in *Klf4*^*ΔPod*^ mice. This also illustrates that preventing the rapid loss of podocytes eliminates the trigger for parietal epithelial cell activation and proliferation.

The mechanisms whereby rapid podocyte loss triggers parietal epithelial cell activation and proliferation have been previously investigated.^12,13,32,33^ We previously demonstrated that key ligand-receptor interactions between podocytes and parietal epithelial cells, such as FN1–*α*V*β*6, are dependent on KLF4-STAT3 signaling.^13^ Pharmacologic STAT3 inhibition in *Klf4*^*ΔPod*^ mice reduced the glomerular expression of both FN1 and ITGB6, while improving albuminuria and reducing parietal epithelial cell activation and proliferation.^13^ In this study, we improve our understanding of this pathway by demonstrating that the concurrent deletion of *Stat3* specifically in podocytes in *Klf4*^*ΔPod*^ mice also reduces the glomerular expression of FN1 and ITGB6. Interestingly, the podocyte-derived soluble chemokines, macrophage migration inhibitory factor, which was also upregulated in the secretome from *Klf4*-*shRNA* podocytes, along with stromal cell–derived factor 1,^13^ were recently identified to be upregulated in injured podocytes.^34^ These authors also showed increased expression of the migration inhibitory factor/stromal cell–derived factor 1 receptor, CXC chemokine receptor 4, in CD44^+^ parietal epithelial cells, suggesting additional ligand-receptor interactions between podocytes–parietal epithelial cells that might be critical for parietal epithelial cell activation and proliferation.^34^ Whether STAT3 activation is also upstream of these pathways, and the convergence of these pathways with FN1–*α*V*β*6 signaling will be a subject of future investigations. Furthermore, while we focused on STAT3 activation in podocytes, pSTAT3 expression is also increased in activated parietal epithelial cells in *Klf4*^*ΔPod*^ mice and in mice treated with NTS.^12^ Therefore, investigating the role of STAT3 activation in parietal epithelial cells using parietal epithelial cell–specific *Stat3* knockout mice are also needed to clearly determine the effect of inhibiting STAT3 signaling in cells other than podocytes in the glomerulus. Furthermore, it remains unclear the potential effect of inhibiting STAT3 signaling specifically in parietal epithelial cells on overall podocyte survival. Future studies are also necessary to determine the repercussions of inhibiting parietal epithelial cell activation specifically in the setting of rapid podocyte loss on albuminuria and progression of glomerular injury.

Apart from the mitigation of glomerular injury in *Klf4*^*ΔPod*^*Stat3*^*ΔPod*^ mice, it is of importance to note that the significant tubular injury and interstitial inflammation and fibrosis observed in *Klf4*^*ΔPod*^ mice was also effectively attenuated on the concurrent knockdown of podocyte-specific *Stat3*. It is likely that this profound decrease in tubulointerstitial damage is secondary to preserved podocyte number and reduced glomerular injury, as we previously observed in other models.^35^ However, studies investigating the effects of podocyte-specific *Stat3* deletion in murine models of tubular injury would elucidate these mechanisms and potential podocyte-tubular cross-talk further.

Our findings also add to previous literature highlighting the critical role of KLF4 in the podocyte. We recently showed that *KLF4* induction in podocytes abrogates podocyte injury, parietal epithelial cell activation and proliferation in a murine model of crescentic GN.^13^ Another recent report demonstrated that overexpression of *KLF4* in podocytes reduced high-glucose–mediated apoptosis and preserved nephrin expression.^36^ Here, impressively, we demonstrate a near complete rescue of the phenotype observed in *Klf4*^*ΔPod*^ mice on podocyte deletion of *Stat3*, suggesting that the detrimental effects of loss of podocyte-specific *Klf4* are largely dependent on STAT3 activation. While not statistically significant, we did observe that some *Klf4*^*ΔPod*^*Stat3*^*ΔPod*^ mice had a mild increase in albuminuria compared with littermate controls, suggesting possible contributions from STAT3-independent mechanisms or the systemic effects of STAT3 activation driving kidney disease in the setting of loss of podocyte-specific *Klf4*. However, *Klf4*^*ΔPod*^*Stat3*^*ΔPod*^ mice appear nearly indistinguishable from littermate controls regarding their kidney function (serum urea nitrogen and creatinine), podocyte, parietal epithelial cell, and tubular health, but their susceptibility to glomerular disease should be the topic of future studies, especially since knockdown of podocyte-specific *Klf4* on a resistant background (C57BL/6) exacerbates kidney injury in both adriamycin nephropathy^37^ and NTS nephritis.^12^

In our studies, we used constitutive transgenic models for generation of both *Klf4* and *Stat3* knockdown mice. Previous studies show that *Stat3* expression is required for early embryonic development, as total body deletion of *Stat3* is lethal by E8.5^38^; however, this is not observed in podocyte. In the kidney, constitutive, cell-specific (stromal, tubular epithelial, podocyte) *Stat3* knockdown has been studied in mice, with no observed effect on development.^9,39,40^ Furthermore, *Stat3* deletion from various kidney cells has been shown to be largely protective, as knockdown of *Stat3* in both FOXD1+ stromal cells and tubular epithelial cells was protective against kidney fibrosis^39,40^ and *Stat3* knockdown from podocytes mitigated against a murine model of HIV-associated nephropathy (model of collapsing FSGS).^9^ From these studies, it is not known if the kidney protection gained from constitutive *Stat3* knockdown would be maintained if the deletion was induced specifically in adult mice. To address this, future studies should focus on the inducible podocyte-specific knockout of *Stat3* in adult *Klf4*^*ΔPod*^ mice as well as in other models of parietal epithelial cell activation and proliferation, which would be of importance to determine potential therapeutic implications for podocyte-specific targeted STAT3 pathway inhibition, thus eliminating off-target effects from systemic STAT3 inhibition.

Interrogation of existing expression arrays from Nephroseq^14^ demonstrated an enrichment of downstream STAT3 target genes in renal vasculitis and to a lesser extent in DKD and lupus nephritis as compared with other glomerular diseases. While the RNA-seq–based proportional cell type deconvolution of bulk RNA-seq data obtained from the NEPTUNE showed that this enrichment of STAT3 genes was primarily in podocytes, future studies are necessary to determine if this is podocyte-specific enrichment is restricted to subtypes of FSGS with parietal epithelial cell activation.

Collectively, the concurrent knockdown of *Stat3* specifically in podocytes effectively preserved podocyte number, kidney health, and overall survival compared with *Klf4*^*ΔPod*^ mice. Combined with previous studies, this emphasizes the critical role of KLF4-mediated inhibition of STAT3 signaling in maintaining podocyte health.

## Supplementary Material

## Data Availability

All animal experiments were conducted in accordance with the NIH Guide for the Care and Use of Laboratory Animals or an equivalent standard that meets or exceeds the ethical and welfare requirements outlined in the NIH Guide. All protocols were approved by the appropriate institutional animal care and use committee.

## Data Availability Statements

Original data generated for the study are available in a public access repository. All original data, including deidentified patient-level data or individual laboratory data measurements, are included in the manuscript and/or supplemental material. Data Type: Aggregated Data; Published Material. Repository Name: Nephroseq and NEPTUNE repositories.

## Supplemental Material

Supplemental Table 1. Primary antibodies.

Supplemental Table 2. Primers used for real-time PCR.

Supplemental Table 3. Available demographic information from Nephroseq.
