# Supplementary material for: Podocyte-Specific Deletion of STAT3 in Krüppel-Like Factor 4–Related Experimental Podocytopathy
Source: J Am Soc Nephrol. 2025 Sep 2;37(3):490–503. doi: 10.1681/ASN.0000000841 (PMC12935330; doi:10.1681/ASN.0000000841)
Supplement: Supplementary file 1 [file jasn-37-490-s001.pdf]

ASN
American Society of Nephrology
JASN
Journal of the American Society of Nephrology
KIDNEY360
Accessing Our World From Every Angle
CJASN
Clinical Journal of the American Society of Nephrology

ASN Journal Disclosure Form

As per ASN journal policy, I have disclosed any financial relationships or commitments I have held in the past 36 months as included below. I have listed my Current Employer below to indicate there is a relationship requiring disclosure. If no relationship exists, my Current Employer is not listed.

R. Bronstein reports the following:
Employer: Stony Brook University

I understand that the information above will be published within the journal article, if accepted, and that failure to comply and/or to accurately and completely report the potential financial conflicts of interest could lead to the following: 1) Prior to publication, article rejection, or 2) Post-publication, sanctions ranging from, but not limited to, issuing a correction, reporting the inaccurate information to the authors' institution, banning authors from submitting work to ASN journals for varying lengths of time, and/or retraction of the published work.

Name: Robert Bronstein
Manuscript ID: JASN-2025-000525R2
Manuscript Title: Podocyte-Specific Deletion of STAT3 in KLF4-Related Experimental Podocytopathy
Date of Completion: August 4, 2025
Disclosure Updated Date: August 4, 2025

ASN American Society of Nephrology
JASN Journal of the American Society of Nephrology
KIDNEY360 Accessing Our World From Every Angle
CJASN Clinical Journal of the American Society of Nephrology

ASN Journal Disclosure Form

As per ASN journal policy, I have disclosed any financial relationships or commitments I have held in the past 36 months as included below. I have listed my Current Employer below to indicate there is a relationship requiring disclosure. If no relationship exists, my Current Employer is not listed.

V. D'Agati reports the following:
Patents or Royalties: UpToDate Royalties; Textbook Royalties (Heptinstall's Pathology of the Kidney, 8th Edition); and Advisory or Leadership Role: Editorial Board Member, Kidney International.

I understand that the information above will be published within the journal article, if accepted, and that failure to comply and/or to accurately and completely report the potential financial conflicts of interest could lead to the following: 1) Prior to publication, article rejection, or 2) Post-publication, sanctions ranging from, but not limited to, issuing a correction, reporting the inaccurate information to the authors' institution, banning authors from submitting work to ASN journals for varying lengths of time, and/or retraction of the published work.

Name: Vivette D. D'Agati
Manuscript ID: JASN-2025-000525R2
Manuscript Title: Podocyte-Specific Deletion of STAT3 in KLF4-Related Experimental Podocytopathy
Date of Completion: July 31, 2025
Disclosure Updated Date: July 21, 2025

ASN American Society of Nephrology JASN Journal of the American Society of Nephrology KIDNEY360 Accessing Our World From Every Angle CJASN Clinical Journal of the American Society of Nephrology

ASN Journal Disclosure Form

As per ASN journal policy, I have disclosed any financial relationships or commitments I have held in the past 36 months as included below. I have listed my Current Employer below to indicate there is a relationship requiring disclosure. If no relationship exists, my Current Employer is not listed.

C. Estrada reports the following:
Employer: Stony Brook University Medical Center; Northport Veterans Affairs Medical Center

I understand that the information above will be published within the journal article, if accepted, and that failure to comply and/or to accurately and completely report the potential financial conflicts of interest could lead to the following: 1) Prior to publication, article rejection, or 2) Post-publication, sanctions ranging from, but not limited to, issuing a correction, reporting the inaccurate information to the authors' institution, banning authors from submitting work to ASN journals for varying lengths of time, and/or retraction of the published work.

Name: Chelsea C. Estrada
Manuscript ID: JASN-2025-000525R1
Manuscript Title: Podocyte-specific Deletion of STAT3 in KLF4-Related Experimental Podocytopathy.
Date of Completion: July 17, 2025
Disclosure Updated Date: July 17, 2025

ASN American Society of Nephrology
JASN Journal of the American Society of Nephrology
KIDNEY360 Accessing Our World From Every Angle
CJASN Clinical Journal of the American Society of Nephrology

ASN Journal Disclosure Form

As per ASN journal policy, I have disclosed any financial relationships or commitments I have held in the past 36 months as included below. I have listed my Current Employer below to indicate there is a relationship requiring disclosure. If no relationship exists, my Current Employer is not listed.

Y. Gowthaman reports the following:
Employer: Stony Brook University; and Research Funding: NIH/NIDDK, Veterans Affairs.

I understand that the information above will be published within the journal article, if accepted, and that failure to comply and/or to accurately and completely report the potential financial conflicts of interest could lead to the following: 1) Prior to publication, article rejection, or 2) Post-publication, sanctions ranging from, but not limited to, issuing a correction, reporting the inaccurate information to the authors' institution, banning authors from submitting work to ASN journals for varying lengths of time, and/or retraction of the published work.

Name: Yogesh Gowthaman
Manuscript ID: JASN-2025-000525R1
Manuscript Title: Podocyte-specific Deletion of STAT3 in KLF4-Related Experimental Podocytopathy
Date of Completion: July 17, 2025
Disclosure Updated Date: July 17, 2025

ASN American Society of Nephrology | JASN Journal of the American Society of Nephrology | KIDNEY360 Accessing Our World From Every Angle | CJASN Clinical Journal of the American Society of Nephrology

ASN Journal Disclosure Form

As per ASN journal policy, I have disclosed any financial relationships or commitments I have held in the past 36 months as included below. I have listed my Current Employer below to indicate there is a relationship requiring disclosure. If no relationship exists, my Current Employer is not listed.

Y. Guo reports the following:
Employer: Stony Brook University

I understand that the information above will be published within the journal article, if accepted, and that failure to comply and/or to accurately and completely report the potential financial conflicts of interest could lead to the following: 1) Prior to publication, article rejection, or 2) Post-publication, sanctions ranging from, but not limited to, issuing a correction, reporting the inaccurate information to the authors' institution, banning authors from submitting work to ASN journals for varying lengths of time, and/or retraction of the published work.

Name: Yiqing Guo
Manuscript ID: JASN-2025-000525R1
Manuscript Title: Podocyte-specific Deletion of STAT3 in KLF4-Related Experimental Podocytopathy,
Date of Completion: July 25, 2025
Disclosure Updated Date: July 25, 2025

ASN
American Society of Nephrology
JASN
Journal of the American Society of Nephrology
KIDNEY360
Accessing Our World From Every Angle
CJASN
Clinical Journal of the American Society of Nephrology

ASN Journal Disclosure Form

As per ASN journal policy, I have disclosed any financial relationships or commitments I have held in the past 36 months as included below. I have listed my Current Employer below to indicate there is a relationship requiring disclosure. If no relationship exists, my Current Employer is not listed.

J. He reports the following:
Employer: Icahn School of Medicine at Mount Sinai; Consultancy: Boehringer Ingelheim Pharmaceuticals, Inc; Vera Therapeutics; Idorsia Therapeutics; Yingli Pharmaceuticals; Ownership Interest: Renalytix AI; Rila Therapeutics;; Research Funding: Ono Pharmaceuticals; and Advisory or Leadership Role: Associate Editor for Kidney International.

I understand that the information above will be published within the journal article, if accepted, and that failure to comply and/or to accurately and completely report the potential financial conflicts of interest could lead to the following: 1) Prior to publication, article rejection, or 2) Post-publication, sanctions ranging from, but not limited to, issuing a correction, reporting the inaccurate information to the authors' institution, banning authors from submitting work to ASN journals for varying lengths of time, and/or retraction of the published work.

Name: John Cijiang He
Manuscript ID: JASN-2025-000525R1
Manuscript Title: Podocyte-specific Deletion of STAT3 in KLF4-Related Experimental Podocytopathy
Date of Completion: July 16, 2025
Disclosure Updated Date: July 16, 2025

ASN American Society of Nephrology
JASN Journal of the American Society of Nephrology
KIDNEY360 Accessing Our World From Every Angle
CJASN Clinical Journal of the American Society of Nephrology

ASN Journal Disclosure Form

As per ASN journal policy, I have disclosed any financial relationships or commitments I have held in the past 36 months as included below. I have listed my Current Employer below to indicate there is a relationship requiring disclosure. If no relationship exists, my Current Employer is not listed.

J. Kim reports the following:
Employer: Stonybrook Univeristy

I understand that the information above will be published within the journal article, if accepted, and that failure to comply and/or to accurately and completely report the potential financial conflicts of interest could lead to the following: 1) Prior to publication, article rejection, or 2) Post-publication, sanctions ranging from, but not limited to, issuing a correction, reporting the inaccurate information to the authors' institution, banning authors from submitting work to ASN journals for varying lengths of time, and/or retraction of the published work.

Name: Joseph Kim
Manuscript ID: JASN-2025-000525R1
Manuscript Title: Podocyte-specific Deletion of STAT3 in KLF4-Related Experimental Podocytopathy
Date of Completion: July 19, 2025
Disclosure Updated Date: July 19, 2025

ASN American Society of Nephrology | JASN Journal of the American Society of Nephrology | KIDNEY360 Accessing Our World From Every Angle | CJASN Clinical Journal of the American Society of Nephrology

ASN Journal Disclosure Form

As per ASN journal policy, I have disclosed any financial relationships or commitments I have held in the past 36 months as included below. I have listed my Current Employer below to indicate there is a relationship requiring disclosure. If no relationship exists, my Current Employer is not listed.

S. Mallipattu reports the following:
Employer: Stony Brook Medicine; Consultancy: Wildwood Therapeutics, Inc.; L.E.K. Consulting; Dedham Group; Graticule;; Research Funding: Dialysis Clinic Inc.; Patents or Royalties: Krüppel-like factor 15 (KLF15) Small Molecule Agonists in Kidney Disease. US 63/018.247. 2023.; and Advisory or Leadership Role: Clinically Integrated Network, Board Member (Accountable Care Organization, LLC Stony Brook Medicine);.

I understand that the information above will be published within the journal article, if accepted, and that failure to comply and/or to accurately and completely report the potential financial conflicts of interest could lead to the following: 1) Prior to publication, article rejection, or 2) Post-publication, sanctions ranging from, but not limited to, issuing a correction, reporting the inaccurate information to the authors' institution, banning authors from submitting work to ASN journals for varying lengths of time, and/or retraction of the published work.

Name: Sandeep K. Mallipattu
Manuscript ID: JASN-2025-000525R1
Manuscript Title: Podocyte-specific Deletion of STAT3 in KLF4-Related Experimental Podocytopathy
Date of Completion: July 17, 2025
Disclosure Updated Date: March 18, 2025

ASN
American Society of Nephrology
JASN
Journal of the American Society of Nephrology
KIDNEY360
Accessing Our World From Every Angle
CJASN
Clinical Journal of the American Society of Nephrology

ASN Journal Disclosure Form

As per ASN journal policy, I have disclosed any financial relationships or commitments I have held in the past 36 months as included below. I have listed my Current Employer below to indicate there is a relationship requiring disclosure. If no relationship exists, my Current Employer is not listed.

D. Salant reports the following:
Employer: Boston University Medical Center; Consultancy: Visterra, UpToDate; Research Funding: NIH; Honoraria: Several academic institutions and national societies; Patents or Royalties: Patent: "Diagnostics in membranous nephropathy" - Boston Medical Center; Advisory or Leadership Role: Editorial board: JASN, Am J Physiol.; Scientific Advisory Board: NEPTUNE; and Other Interests or Relationships: National Kidney Foundation Medical Advisory Board.

I understand that the information above will be published within the journal article, if accepted, and that failure to comply and/or to accurately and completely report the potential financial conflicts of interest could lead to the following: 1) Prior to publication, article rejection, or 2) Post-publication, sanctions ranging from, but not limited to, issuing a correction, reporting the inaccurate information to the authors' institution, banning authors from submitting work to ASN journals for varying lengths of time, and/or retraction of the published work.

Name: David J. Salant
Manuscript ID: JASN-2025-000525R1
Manuscript Title: Podocyte-specific Deletion of STAT3 in KLF4-Related Experimental Podocytopathy
Date of Completion: July 16, 2025
Disclosure Updated Date: July 16, 2025
