# Supplementary material for: Podocyte-Specific Deletion of STAT3 in Krüppel-Like Factor 4–Related Experimental Podocytopathy
Source: J Am Soc Nephrol. 2025 Sep 2;37(3):490–503. doi: 10.1681/ASN.0000000841 (PMC12935330; doi:10.1681/ASN.0000000841)
Supplement: Supplementary file 2 [file jasn-37-490-s002.pdf]

Podocyte-Specific Deletion of STAT3 in KLF4-Related Experimental Podocytopathy

Yogesh Gowthaman[1], Chelsea C. Estrada[1,2], Joseph Kim[1], Yiqing Guo[1], Robert Bronstein[1], David J. Salant[3], John C. He[4], Vivette D. D'Agati[5], Sandeep K. Mallipattu[1,2]

[1]Division of Nephrology, Department of Medicine, Stony Brook University, Stony Brook, NY
[2]Division of Nephrology, Department of Medicine, Northport Veterans Affairs Medical Center, Northport, NY
[3]Division of Nephrology, Department of Medicine, Boston University School of Medicine, Boston, MA
[4]Division of Nephrology, Department of Medicine, Icahn School of Medicine at Mount Sinai, NY, NY
[5]Department of Pathology and Cell Biology, Columbia University, NY, NY

Supplemental Table of Contents:

Supplemental Table 1: Primary Antibodies
Supplemental Table 2: Primers used for real-time PCR
Supplemental Table 3: Available Demographic Information from Nephroseq

Supplemental Table 1: Primary Antibodies

Antibody	Species	Company	Catalog number	Dilution Factor
Phospho-Stat3	Rabbit	Cell Signaling Technologies	9145S	1:100
Synaptopodin	Rabbit	Sigma	SAB3500585	1:200
WT1	Mouse	Santa Cruz Biotechnology	SC-7385	1:50
CD44	Rat	BioLegend	103001	1:100
AKAP12	Rabbit	Proteintech	25199-1-AP	1:100
Ki67	Rat	Biolegend	652402	1:100
FN1	Rabbit	Millipore Sigma	AB2033	1:100
ITGB6	Rabbit	Sigma	HPA023626	1:100
Vimentin	Rabbit	Cell Signaling	5741S	1:100
α-SMA	Mouse	Sigma	9698T	1:10000
GR1	Rat	BioRad	MCA2387	1:100
F4/80	Rat	eBioscience	14-4801-85	1:100

Supplemental Table 2: Primers used for real-time PCR

Gene	Forward primer	Reverse primer
Mouse *Stat3*	CTACTGCGCTTCAGCGAGAGCAGC	GTCTTCAGGTACGGGGCAGCAC
Mouse *Il-6*	CAAAGCCAGAGTCCTTCAGAG	GCCACTCCTTCTGTGACTCC
Mouse *Socs3*	CCTGGTGTTTCCAAGGAAGT	CTTTCCACAGGGATGAGGTT
Mouse *Icam-1*	GTGATGCTCAGGTATCCATCCA	CACAGTTCTCAAAGCACAGCG
Mouse *Synpo*	CTTTGGGGAAGAGGCCGATTG	GTTTTCGGTGAAGCTTGTGC
Mouse *Wt1*	CAGGCTGCAATTAAGAATATTTTTT	GAAGTCACCACTTGGTTATGGTTTTCT
Mouse *Cd44*	AGCAGCGGCTCCACCATCGAGA	TCGGATCCATGAGTCACAGTG
Mouse *Cd74*	ATGACCCAGGACCATGTGATG	CCCTTCAGCTGCGGGTACT
Mouse *Ccnd1*	CACCAACGCACTTTCCTTTTCTTTTTCCA	TCCCAGAAGGGCTTTTTCAATTCTG
Mouse *Fn1*	ATGGTACAGCTGATCCTGCC	GCCCTGGTTTGTACCTGCTA
Mouse *Itgb6*	TTGGCCACCTTCTGCCCAAAGACT	TTTCTGTCTGGGCTCACGTC
Mouse *Lcn2*	AATGTCACCTCCATCCTGGTC	GCCACTTGCACATTGTAGCTC
Mouse *Havcr1*	ACGGCTCTCTCCTAACTGGT	CCACCACCCCCTTTACTTCC

Supplemental Table 3: Available Demographic Information from Nephroseq [1]

Disease	Sample Size	Age (Mean ± SD)	eGFR (Mean ± SD)	Male (%)
Control	21	47.1 ± 11.7	105.4 ± 30.9	12 (57.1)
HTN	15	57.1 ± 11.7	40.8 ± 23.8	12 (80)
DKD	12	52.9 ± 30.3	54.7 ± 10.9	8 (66.6)
FSGS	25	45.8 ± 15.2	74.4 ± 33.2	13 (52)
IgA Nephropathy	27	36.4 ± 14.0	73.9 ± 37.0	19 (70.3)
Lupus Nephritis	32	35.0 ± 13.0	63.7 ± 28.9	7 (21.8)
MCD	14	34.9 ± 17.3	106.4 ± 49.8	8 (57.1)
Vasculitis	23	58.4 ± 13.8	45.9 ± 30.8	13 (56.5)

HTN: Hypertension, DKD: Diabetic Kidney Disease, FSGS; Focal Segmental Glomerulosclerosis, MCD: Minimal Change Disease

REFERENCES:

1. Ju W, Greene CS, Eichinger F, et al. Defining cell-type specificity at the transcriptional level in human disease. *Genome Res*. Nov 2013;23(11):1862–1873. doi:10.1101/gr.155697.113
